# Variability on the energy properties of charcoal and charcoal briquettes for barbecue

**DOI:** 10.1016/j.heliyon.2022.e10052

**Published:** 2022-08-01

**Authors:** A. Mencarelli, R. Cavalli, R. Greco

**Affiliations:** Department of Land, Environment, Agriculture and Forestry, University of Padova, Viale dell’Università 16, Legnaro (PD), 35020, Italy

**Keywords:** Charcoal, Charcoal briquettes, Charcoal properties, Proximate analysis, Barbecue, Heating value, Biofuel, Grilling

## Abstract

In recent years there has been a strong increase in interest in the world of barbecues and outdoor cooking in high-income countries. Referring to FAO data, an exponential growth in imports of charcoal was observed in Europe and North America. Italy is one of the major European consumers and importers. On the market it is possible to find material with different characteristics and origins. However, analysis aimed at ascertaining the quality of the material are poorly performed. This research aimed to analyze the energy properties of charcoal commonly available on the Italian market. Twenty-four bags of charcoal and charcoal briquettes were analyzed. Eighteen samples represent the products most easily found on the market, in stores and on websites. In addition, six samples were supplied directly by the producer/importing company. The samples were grouped according to the continent of origin of the material (Europe, North-Central America and South America). Charcoal briquette samples were included together in a group. Referring to the ISO 17225-1 standard, the moisture content, ash content, heating value, volatile matter and fixed carbon were determined. Except for the moisture content, the results of the tests performed on all parameters show a strong variability both between different groups and within the same group. In detail, the European charcoal samples show characteristics more suitable for their use in barbecues. These have the highest values of fixed carbon and heating value and, at the same time, low values of ash and volatile matter. On the contrary, charcoal briquettes have less suitable characteristics for barbecue. The work also highlighted some gaps in the reference standard relating to laboratory analyses. To ensure careful control of the qualitative characteristics of the products on the market, it is necessary to promote the creation of a quality brand.

## Highlights

• Absence of information on the characteristics of the charcoal on the market.
• Properties influenced by origin, wood species and production process.
• Presence of gaps in reference standards relating to laboratory analysis.
• High variability of the energy properties of the charcoal and briquettes.
• Poor representativeness of the HHV_0_ determined as indicated by the standard.

## 1 Introduction

Charcoal has always played a fundamental role as a fuel in human history. Even today it is widely exploited in several countries, with uses ranging from domestic use for cooking food to industrial applications [1, 2]. Its importance is underlined by the FAO indicating that about 17% of the wood resources used as fuel on a global scale are transformed into charcoal [3]. Indeed, charcoal is a fuel of primary importance as it plays a key role as a source of energy for urban areas in many low- and middle-income countries [4]. In these countries, it is commonly used for cooking food and domestic heating [5, 6]. Moreover, charcoal production represents a valuable resource for the local populations of these areas guaranteeing economic as well as energy support [7]. However, its production, use and export can cause several and serious repercussions on fragile local ecosystems due to the often-indiscriminate cutting of forest areas and the polluting emissions released during its use [3, 8, 9]. The use of charcoal is also widespread in high-income countries for cooking food, due to the characteristics it gives to food in terms of flavor and texture [10]. The average annual consumption of charcoal in Europe is around one million tons, however only a small part of this quantity is produced on the continent [11]. In Italy, almost all the charcoal commonly available on the market comes from foreign countries. Most of the material arriving in the country comes from Eastern European countries, such as Croatia or Bosnia-Herzegovina, but even more from non-EU countries, mainly from Latin America (Argentina, Cuba, Paraguay and Venezuela) or from African countries (Nigeria) [12].

Referring to the annual average production data provided by FAO for the period between 2000 and 2019 [13], the world scene is dominated by countries such as Brazil (644 kt/year), Nigeria (388 kt/year) and Ethiopia (371 kt/year). However, much of the production of these nations is aimed at satisfying their internal demand. For example, Brazil allocates most of this fuel to the iron industry rather than for domestic uses [14]. Therefore, in relation to international exports, there are other countries that excel. Among these are, for example, Indonesia (255 kt/year), Paraguay (127 kt/year) and Argentina (98 kt/year). In the last few years, the significant increase in interest in the barbecue sector has led to a massive increase in exports of charcoal to high income countries where internal production does not meet the growing demand [3]. Among these, for example, there are several European countries, including Germany (182 kt/year), France (72 kt/year) and the United Kingdom (71 kt/year) but also countries such as the United States (69 kt/year) and Japan (144 kt/year). Among the fifteen main global importers of charcoal there is also Italy whose average import rate, for the period considered, is 59 kt/year. The country's import level trend and the inland consumption are growing, in 2019 a consumption of 72 kt was estimated [15].

Charcoals with different characteristics and qualities are easily available on the market. There are many factors that affect the quality of the material. Among the most important it is necessary to consider the variability due to the different countries of production, which forest trees and parts of them are used, the carbonization process (e.g., charcoal pile or brick kiln) and its efficiency, all together affect the quality of the charcoal [16]. In particular, the production process has a strong impact on the properties of the fuel. The final carbonization temperature and the heating rate, affect the characteristics of the material [17]. The same kiln used can produce different qualities of charcoal depending on the combination of the starting material and the carbonization process [18]. Furthermore, in addition to lump charcoal, the use of charcoal briquettes is increasing. This product represents a particularly widespread fuel used for barbequing and cooking in many high-income countries such as United States, Europe, Australia and Japan [19]. The briquetting of the charcoal allows to improve the characteristics of the material in terms of density and burning times [4]. Starting from the charcoal powder, several types of additives are added, for example starch, which together with water allow the binding of the material [20].

Despite the widespread diffusion and growing demand for barbecue charcoal, analyses conducted by producers or sellers aimed at verifying the quality characteristics of the material are scarcely widespread. The data, as well as the analysis reports, are not easily available to end consumers with the consequent possibility of purchasing material of uncertain quality. Performing analysis could help to identify charcoals with characteristics unsuitable for barbecue uses, determining repercussions both on human health and on the environment. The qualitative parameters to be determined are those reported in the reference standard, the EN ISO 17225–1, in detail Table 14—*Specifications and classification of charcoal* [21].

In literature, few studies analyzed the qualitative characteristics of charcoal commonly available on the market, focusing only on a few samples and on local charcoal. Dias Júnior et al. [22], analyzed various charcoal commonly available on the Brazilian market. These products were almost all made from eucalyptus wood and produced in Brazil, limiting the variability of the samples in terms of wood species and origin of the material. Huang et al. [23] determined the characteristics of 10 charcoals and briquettes commonly used in Taiwan, produced in China, Indonesia and Taiwan. Kajina et al. [24] analyzed the charcoal characteristics produced using different biomasses in Thailand. As regard the European market, no studies have been conducted. In this context, the variability of the products on the market is high since most of the material is imported from different continents. The scope of this work is to analyze the main energy properties of the charcoal commonly found on the Italian market.

## 2 Materials and methods

### 2.1 Samples analyzed

To conduct an analysis on the charcoal commonly available on the market, both samples of charcoal and charcoal briquettes were used. The samples were purchased from large-scale retailers, shops and specialized internet sites for barbecues or supplied directly by the importing company or the producer. A total of twenty-four different bags of charcoal and charcoal briquettes, representative of the products most commonly found on the market in Italy, were analyzed. Of these, four bags were purchased on specialized websites, seven from specialized barbecue shops, seven from large-scale retail stores. Three bags were supplied directly by the charcoal producer and finally three supplied by the importer company. Each sample was different in terms of country of production, tree species and carbonization process used. Referring to Table 1, the charcoal samples were grouped into groups based on the continent of origin. As regards the samples of charcoal briquettes, being fewer than the previous ones, they were grouped into a single group without making distinctions based on their origin. Overall, twenty-four samples were analyzed, identified by a code and a progressive number. Currently producers and distributors are not obliged to report information in the bag relating to which wood species was used and which country or continent the material comes from [25]. Therefore, due to the lack of information, for some products available on the market it is not easy to trace the information previously reported [26]. Overall, the most difficult information to find is relating to the production process used, which is almost never reported on the bags and often not even known by the importing companies. Regarding the countries of production and the wood species used, there is a greater availability of information for charcoal of European and Central-North American origin. Some of these samples are accompanied by FSC® sustainability certification facilitating traceability of product characteristics. On the contrary, for several South American samples there was a lack of information regarding the wood species used with the danger of using protected tropical species [12].

**Table 1:** Country of production, type of product, wood species and carbonization process of all samples.

Sample Identification	Continent of origin	Production country	Product type	Wood species	Carbonization process
EU01	Europe	EU	Charcoal	Birch (*Betula spp.*) and aspen (*Populus tremula*)	Unknown
EU02	Europe	EU	Charcoal	Birch (*Betula spp.*) and oak (*Quercus spp.*)	Unknown
EU03	Europe	Ukraine	Charcoal	Common ash (*Fraxinus excelsior*), common oak (*Quercus robur*) common hornbeam (*Carpinus betulus*) Beech (*Fagus sylvatica*)	Unknown
EU04	Europe	Ukraine	Charcoal	Common hornbeam (*Carpinus betulus*)	Unknown
EU05	Europe	Italy	Charcoal	Holm oak (*Quercus ilex*)	Charcoal pile
EU06	Europe	Italy	Charcoal	Manna ash (*Fraxinus ornus*) and European hop-hornbeam (*Ostrya carpinifolia*)	Charcoal pile
EU07	Europe	Italy	Charcoal	Beech (*Fagus sylvatica*), European hop-hornbeam (*Ostrya carpinifolia*), common hornbeam (*Carpinus betulus*), manna ash (*Fraxinus ornus*) and hazel (*Corylus avellana*)	Charcoal pile
EU09	Europe	Croatia	Charcoal	Beech (*Fagus sylvatica*), hornbeam (*Carpinus spp.*) and oak (*Quercus spp.*)	Metal kiln
EU10	Europe	Poland	Charcoal	Beech (*Fagus sylvatica*)	Unknown
EU11	Europe	Finland	Charcoal	Birch (*Betula spp.*)	Unknown
CNA14	Central-North America	USA	Charcoal	Hickory (*Carya spp.*), maple (*Acer spp.*) and oak (*Quercus spp.*)	Unknown
CNA15	Central-North America	USA	Charcoal	Oak (*Quercus spp.*) and ebony (*Diospyros spp.*)	Unknown
CNA16	Central-North America	Mexico	Charcoal	Mesquite (*Prosopis spp.*), oak (*Quercus spp.*) and pecan (*Carya illinoinensis*)	Unknown
CNA17	Central-North America	Cuba	Charcoal	Marabù (*Dichrostachys cinerea*)	Unknown
SA18	South America	Argentina	Charcoal	Quebracho	Unknown
SA20	South America	Argentina	Charcoal	Unknown	Unknown
SA21	South America	Venezuela	Charcoal	Carob (*Ceratonia siliqua*), mesquite (*Prosopis spp.*) and oak (*Quercus spp.*)	Charcoal pile
SA22	South America	Argentina	Charcoal	Unknown	Unknown
SA23	South America	Argentina	Charcoal	Unknown	Unknown
SA24	South America	Argentina	Charcoal briquettes	Quebracho blanco (*Aspidosperma quebracho-blanco*)	Unknown
B08	Europe	EU	Charcoal briquettes	Beech (*Fagus sylvatica*)	Unknown
B12	Europe	EU	Charcoal briquettes	Birch (*Betula spp.*) beech (*Fagus sylvatica*), hornbeam (*Carpinus spp.*) and oak (*Quercus spp.*)	Unknown
B13	Asia	Sri Lanka	Charcoal briquettes	Coconut palm (*Cocus nucifera*)	Unknown
B19	South America	Argentina	Charcoal briquettes	Quebracho colorado (*Schinopsis lorentzii*), guaiac (*Guaiacum officinale*) and carob (*Ceratonia siliqua*)	Brick kiln

### 2.2 Analysis conducted

Analysis were performed with reference to the provisions of the specific technical standard for charcoal EN ISO 17225–1:2014 - *Specifications and classification of charcoal* [21]. The determination of each parameter was conducted following the provisions of the specific reference standards. To obtain representative values of the characteristics of the material, three repetitions for each parameter investigated were performed for all twenty-four samples as required by the respective EN ISO standards and to have enough values to conduct a statistical analysis. The repeatability of the tests was validated as indicated by the specific standards. The analyzed parameters are heating value, moisture content, ash content, volatile matter and fixed carbon (Table 2). The analysis conducted initially required a sample preparation phase. This is to have a homogeneous sample, of suitable size and which reflects the overall properties of the material to standardize the procedures and methods of analysis. Therefore, the charcoal samples were ground using a knife mill preparing the material for the subsequent analyses as reported in EN ISO 14780:2017 standard [27].

**Table 2:** Analyzes conducted on the material as required by EN ISO 17225–1: 2021 Table 14.

Parameter	Unit of measure	Reference standard
Moisture content	% ar	EN ISO 18134–1:2015 [30]
		EN ISO 18134–2:2017 [31]
Ash content	% db	EN ISO 18122:2015 [32]
Volatile matter	%	EN ISO 18123:2015 [33]
Fixed carbon	% db	EN ISO 17225–1:2021 Table 14 [21]
Heating value	MJ/Kg	EN ISO 18125:2017 [28]

a.r. as received.

d.b. dry basis.

As regards the heating value, due to the low homogeneity of charcoal, the value obtained following the reference standard, the EN ISO 18125:2017 [28], may not sufficiently reflect the energy variability found in the material during its use. The lack of homogeneity in the sample linked to the origin, species of trees and parts of this used, but also the carbonization system and the relative result affect the determined values which may therefore not be representative. To obtain a value that better reflects the characteristics of the product, the parameter was also determined following a laboratory procedure developed by the same authors specifically for charcoal [29]. This, unlike the standard method, involves the use of unground sub-samples, weighing 0,5 g, taken from single elements that differ macroscopically for the trees and their components used (for example stems with or without bark, and twigs) as well as for the result of the production process (efficient or not). The presence or absence of parts of the product that are not completely carbonized allows to evaluate the efficiency of the production process. A greater presence of portions of product that are scarcely carbonized determine a lower efficiency of the production process. Therefore, in the developed procedure, the sample is neither grinding nor pressing. In this way these can be used as they are, verifying any incongruities in the internal energy properties of the same material belonging to the same bag. The calorimetric analysis is performed using the same calorimetric bomb used in the standard procedure. At the same time the parameter was analyzed following the provisions of the EN ISO 18125:2017 standard [28].

### 2.3 Statistical analysis

Statistical analysis was performed using Statgraphics 19 software. The data collected in the laboratory tests were subjected to the one-way ANOVA statistical analysis to test the presence or absence of differences among the sample averages considering the respective variances. The parameters analyzed were compared based on the continent of origin of the sample (Europe, Central-North America and South America) and the type of product (charcoal as it is and briquettes). The multiple range test used to define which samples averages are statistically different from the others was the Tukey's HSD test.

## 3 Results and discussion

Based on the results of the energy properties performed, a high variability was observed both between the individual samples belonging to the same group and between the different groups. Table 3 shows the results of the analysis of variance for all the parameters analyzed. The values obtained in this study were compared with the data reported in similar works in literature.

**Table 3:** Analysis of variance of all the parameters analyzed.

Parameter		S.S.	D.F.	M.S.	F-Ratio	P-value
Moisture content (M)	Between groups	302.4	23	13.2	18.4	0.00
	Within groups	34.4	48	0.7		
	Total	336.8	71			
Ash content (A)	Between groups	2495.1	23	108.5	443.7	0.00
	Within groups	11.7	48	0.3		
	Total	2506.8	71			
Volatile matter (VM)	Between groups	4513.7	23	196.3	366.0	0.00
	Within groups	25.7	48	0.5		
	Total	4539.5	71			
Fixed carbon (FC)	Between groups	9574.8	23	416.3	776.1	0.00
	Within groups	25.8	48	0.5		
	Total	9600.6	71			
Heating value (standard method) (HHV_0_)	Between groups	770.3	23	33.5	109.1	0.00
	Within groups	14.7	48	0.3		
	Total	785.1	71			
Heating value (proposed method) (HHV_0_)	Between groups	1074.3	23	46.7	75.3	0.00
	Within groups	29.8	48	0.6		
	Total	1104.1	71			

S.S. Sum of Squares.

D.F. Degree of Freedom.

M.S. Mean Square.

### 3.1 Moisture content

**Figure 1:**
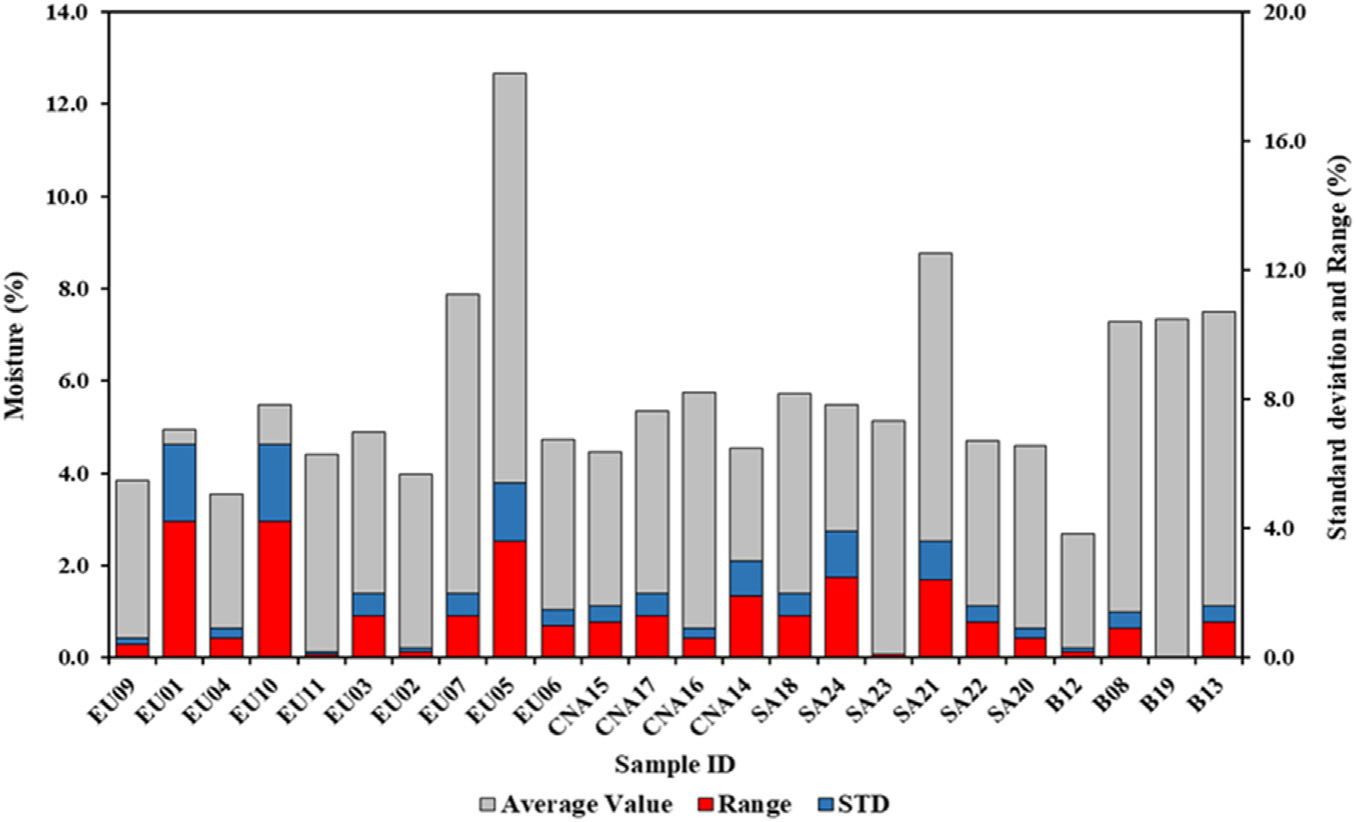
Average moisture content, standard deviation and range of variations of all samples analyzed.

This parameter is the only one where less variability was observed (Figure 1). In detail, the European group presents an average value of the ten analyzed samples of 5.6%. Except for sample EU05, for which an average value of 12.7% was determined, the remaining samples show average values below 8.0% with limited internal variability. The Central-North American samples, although less numerous, have a lower average value than the previous group, equal to 5.0% and less variability. The South American samples showed a mean group value of 5.7%, in line with the values of the previous groups. Also, in this case the presence of a high value was found. The sample SA21 indeed has an average content of 10.0%. About charcoal briquettes, except for sample B12 (2.6%), the samples have an average value of around 7.0%. A greater value of moisture content in the briquettes, more than 9.0%, was also observed by Dias Júnior et al. [22]. In the briquettes samples of Huang et al. [23] the moisture content was approximately between 3.0 and 7.0%. Therefore, overall, the moisture content of the charcoal briquettes samples is higher than what observed for the charcoal groups as it is. A higher value of this parameter is due to the necessity during the processing of the material to have a higher moisture content to facilitate compression as it occurs for other woody biofuels such as pellets [20]. In fact, if the material is excessively dry it would tend to discard more easily. During the production of the charcoal briquettes, the moisture content has values of about 20% but with the possibility of reaching values around 30% and then undergoing a drying process following the compression [34, 35]. With regards to the charcoal as it is, the samples have an average value of around 6.0%. A low humidity value is due to the pyrolysis process used to make the charcoal during which the initial humidity of the material used is lost [36]. Furthermore, in charcoal the hygroscopicity is reduced due to carbonization process. This depends on the final temperature of the carbonization process, increasing the carbonization temperature the moisture adsorbed by the charcoal decreases [37].

### 3.2 Ash content

**Figure 2:**
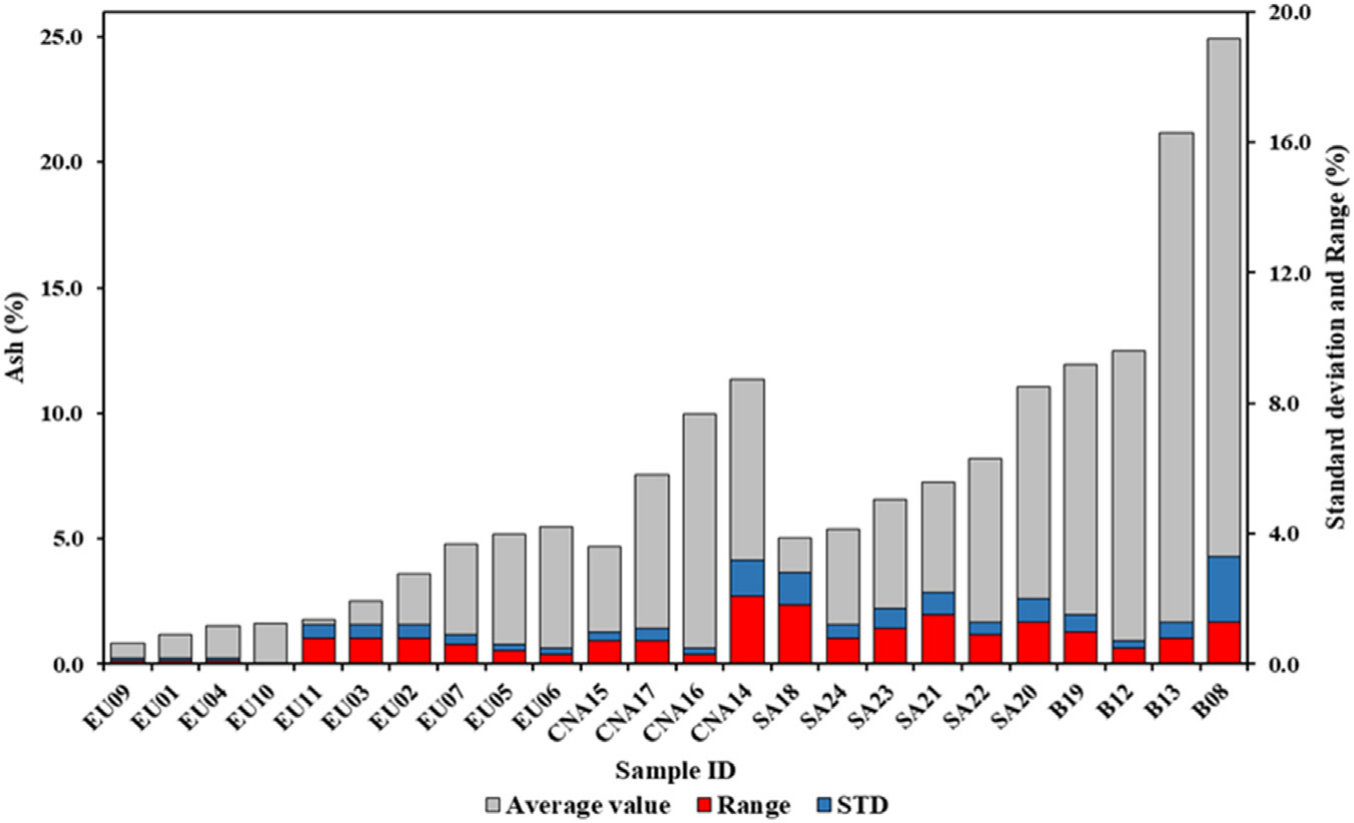
Average ash content, standard deviation and range of variations of all samples analyzed.

This is one of the main important parameters for barbecue consumers. This parameter shows a high degree of variability between the different groups of samples analyzed with statistically significant differences even within the same group (Figure 2). The variability in terms of ash content was also observed in other works. Dias Júnior et al. [22] found ash values ranging between 0.5% and 2.5%. Similar values were found by Kajina et al. [24] (1.6–4.7%) while Huang et al. [23] obtained nonhomogeneous values, ranging between 2.0% and 20.0%. Regarding this study the European samples has a tendentially lower ash content with an average value of the group of 2.9%. The American samples, both as regards the Central-North American and South American groups, show higher values equal to 8.4% and 7.3% respectively. The differences found between the different groups are due to the use of different tree species and their components (e.g., stem with bark or branches) but also to the different carbonization processes used. The composition of the material influences this parameter [1] as well as the heating rate and the temperature of the carbonization process [17]. The presence of soil, dirt or other contaminants can contribute to the increase of this parameter. The average ash content for charcoal briquettes samples is significantly higher than what observed for charcoal as it is. The average value of the group is 17.6%. High values of ash in briquettes, higher than 20%, have also been found in other papers [22, 23]. The presence of such a high value is due to the use of binding agents added to this product, for example corn starch or clay, needed to improve the compaction of the briquette and that may represent up to 5.0% of the material [4, 34, 38]. A high ash content in charcoal briquettes can represent a serious problem during their use both for the environmental repercussions and potentially also for human health due to the inhalation of fine dust [39]. In addition, the high ash content charcoal causes the need for frequent cleaning and frequent maintenance of barbecues with the possibility of forming layers of ash above the embers that could transfer to the food [40].

### 3.3 Volatile matter

**Figure 3:**
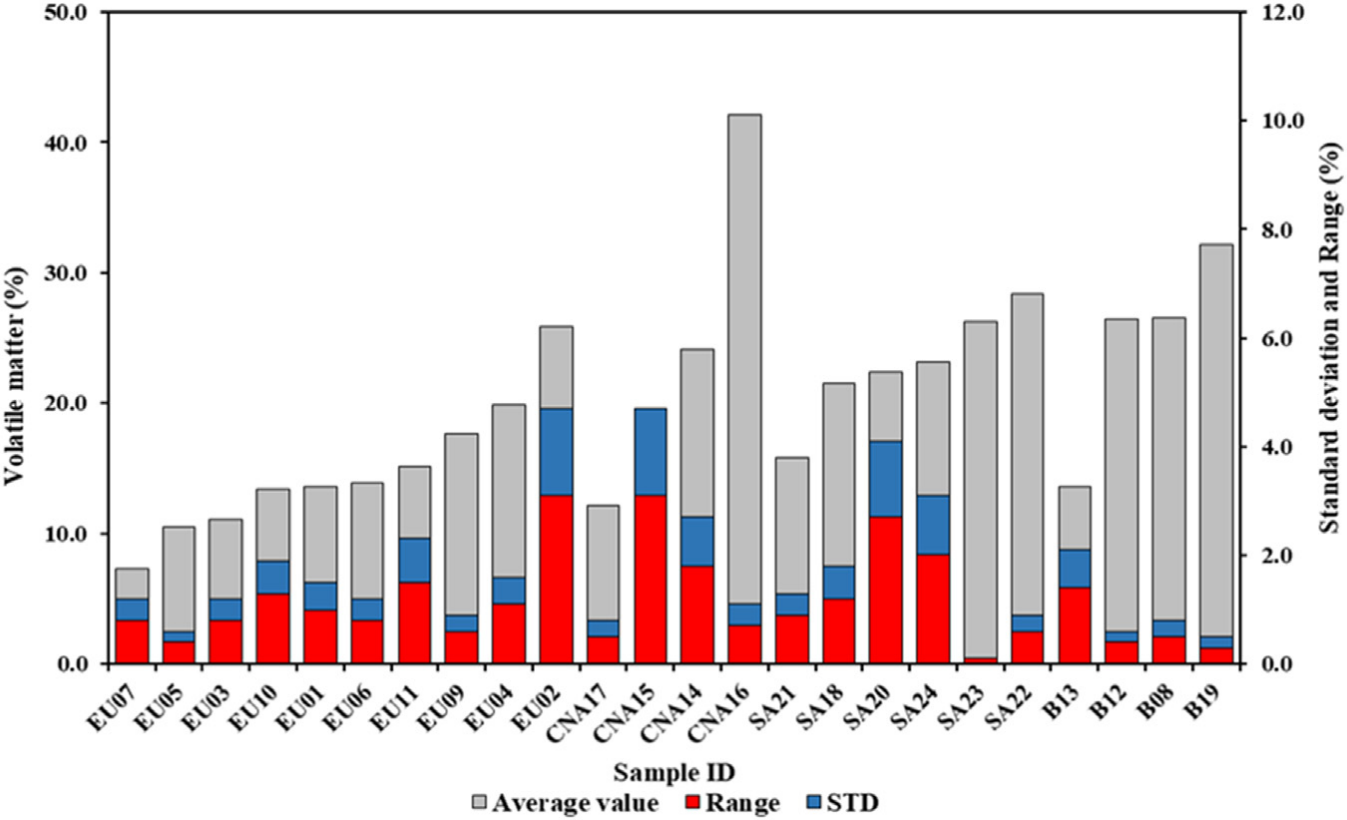
Average volatile matter content, standard deviation and range of variations of all samples analyzed.

Also for this parameter was observed a great variability between the groups (Figure 3). These differences are due to the characteristics of the samples in terms of wood species and conditions of production process used. Also, in Dias Júnior et al. [22] nonhomogeneous values were observed, ranging between 15.0% and 35.0% in charcoal and between 10.0% and 20.0% in charcoal briquettes. In the samples of Huang et al. [23], both in briquettes and in charcoal, values ranging from 20.0-30.0% were determined. The average value found for this parameter in the European group is equal to 14.8%. The average content of volatile matter found for the Central-North American group, equal to 24.5%, is significantly higher than previously determined. A high variability was also found in this case between the different samples. The group of South American samples has an average value of 22.9%, like what was found for the previous group but higher than the European one. Finally, the charcoal briquettes have an average value of the group, equal to 24.7%, comparable to that observed in the American samples. Overall, considering the different numbers in terms of samples within each group, the European samples tend to have a lower content of volatile matter than what was found for the other groups. The production process influences this parameter; in fact, an increase in the rate of heating determines a reduction in volatile matter, because most of them are removed [18]. Charcoal with highly volatile matter can be easily ignited but could cause a combustion with a lot of smoke, while charcoal presenting low values of this parameters could be difficult to ignite but burn much more regularly [38].

**Figure 4:**
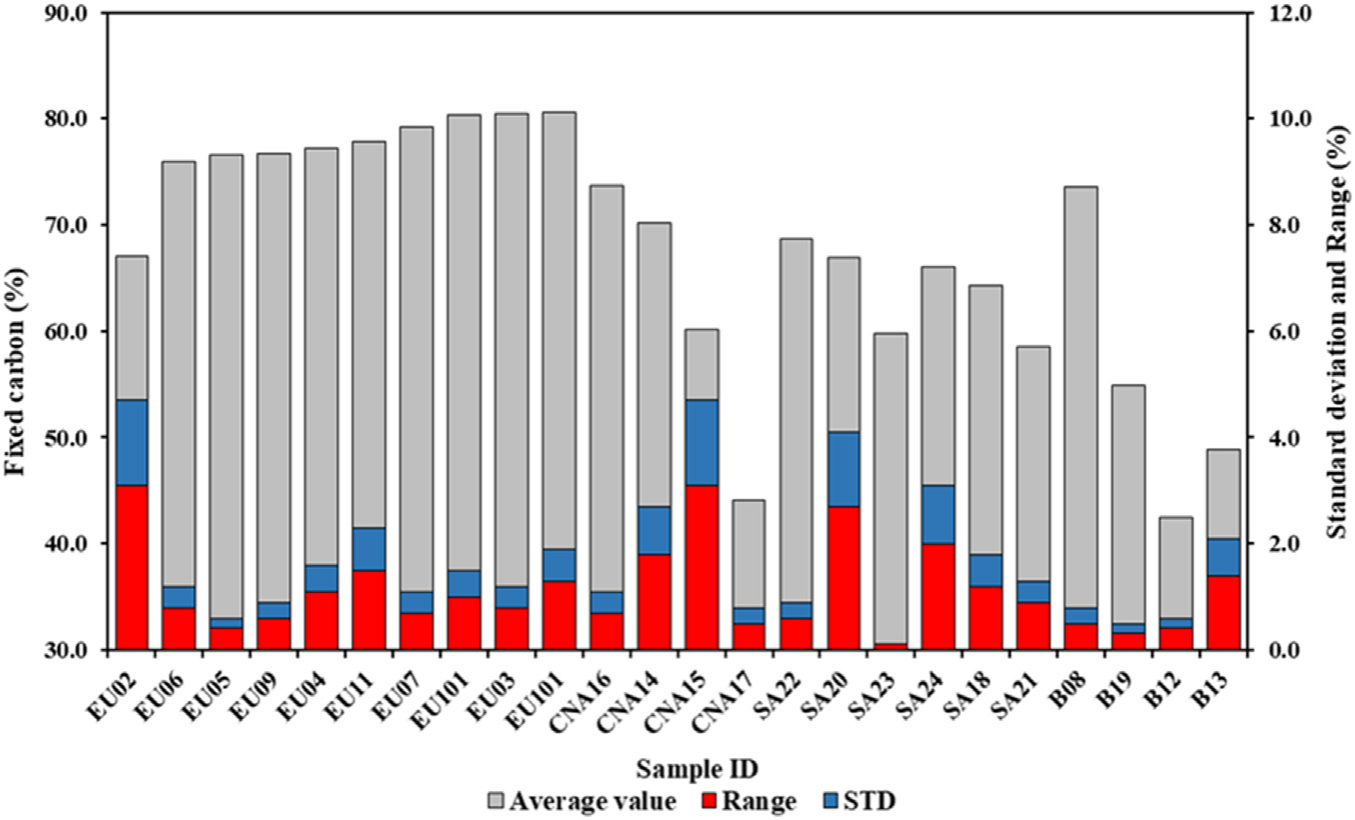
Average fixed carbon content, standard deviation and range of variations of all samples analyzed.

### 3.4 Fixed carbon

The three previous parameters have repercussions on the fixed carbon value. A higher fixed carbon value is due to lower moisture content, ash and volatile matter content. On the contrary, when the moisture content, volatile matter and ash have high values, the fixed carbon content is lower. The latter one is also influenced by the carbonization temperature to which the material has been subjected, an increase in temperature determines an improvement of the fixed carbon, thus reaching higher values, and a decrease in volatile matter [41]. This parameter is on average higher for the European samples than for the South American and Central-North American samples (Figure 4). The average fixed carbon content for the European samples is 77.2%. Excluding the sample EU02 which has a much lower value than the others (67.0%), the samples show a variability that is not particularly accentuated, oscillating in terms of average value around 75.0% and 80.0%. The North-Central American group has an average value of 62.0%. The minimum found belongs to the sample CNA16 with a value of 43.8% while the sample CNA17 has a higher absolute value, respectively of 74.0%. The average fixed carbon value of the South American samples is 64.1%. This value is slightly higher than what found for the previous group but still lower than the European one. In this case the minimum observed value is 58.2% for the SA20 sample while the absolute maximum is 69.2% for the SA21 sample. Regarding charcoal briquettes, the average value is 55.0%, significantly lower than what was found for charcoal. Lower values than charcoal samples are link to the high ash content, volatile matter and moisture content found for this type of product. In the practical use of charcoal, high values in terms of moisture and ash content, and at the same time fixed carbon present with not too high values, can cause difficulties in igniting the fuel, due to the high moisture content, and at the same time an irregular combustion of the material (low fixed carbon content) as well as the need to frequently remove the combustion residues (high levels of ash) [22]. The briquettes analyzed by Huang et al. [23] have low fixed carbon values, less than 50.0%. Even lower values were determined by Dias Júnior et. al [22] (36.6–43.3%). A barbecue charcoal to be considered of good quality it should be characterized by high values of fixed carbon content, greater than 75% [42]. This threshold, except for the EU02 sample, is only exceeded by the European samples.

### 3.5 Heating value

**Figure 5:**
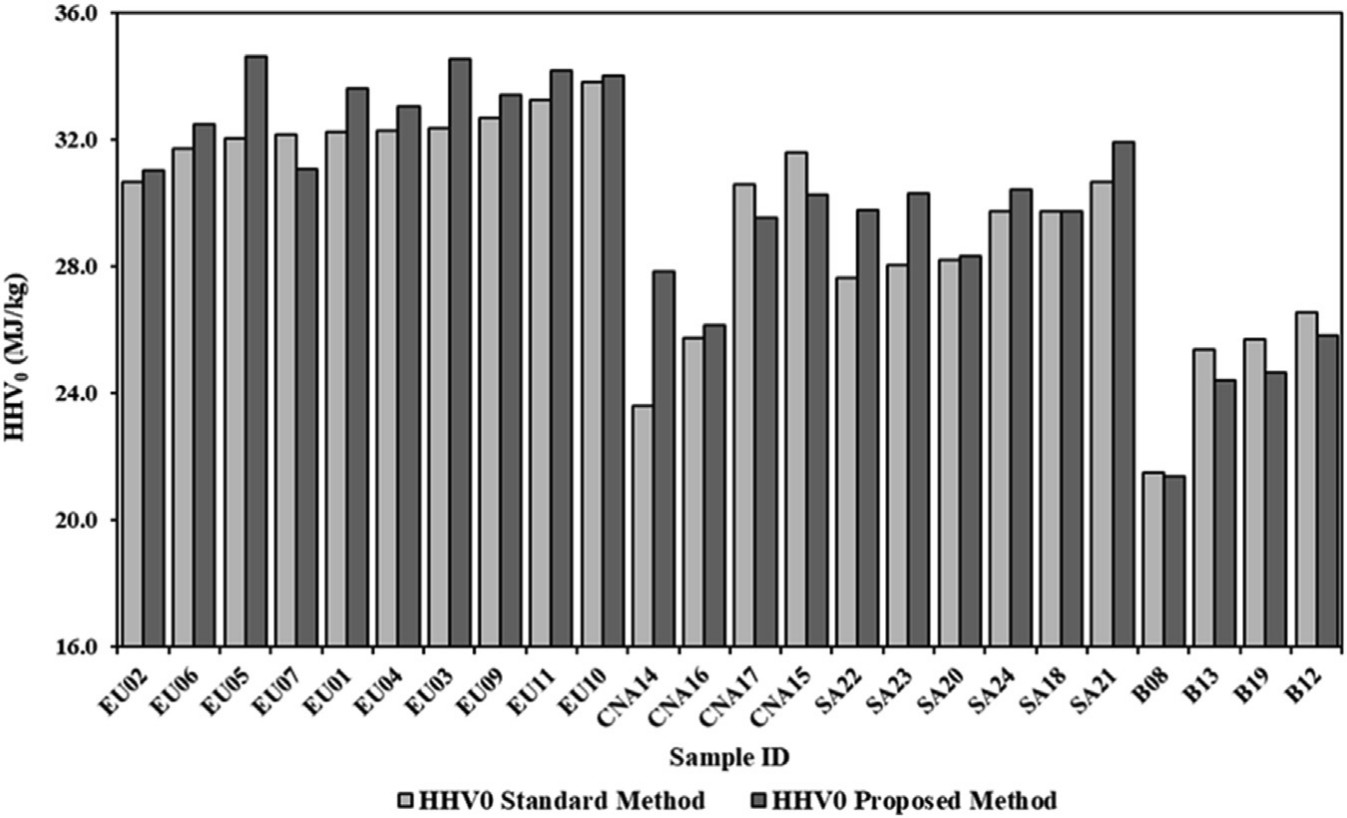
Average HHV_0_ of the samples analyzed using the standard method and proposed method.

This parameter is closely related to the quality of the charcoal; therefore, it is conditioned by the quantity of moisture content, volatile matter and ashes [4]. The higher heating value on an anhydrous basis was used to avoid the influence of the water content on the value of the parameter. Even the heating value shows a high inhomogeneity, both following the method indicated by the standard and the proposed method (Figure 5). The European samples tend to have a higher average HHV_0_, both with the standard method (32.3 MJ/kg) and with the proposed one (33.2 MJ/kg), compared to the group of North American samples (27.9 MJ/kg and 28.5 MJ/kg) and South Americans (29.0 MJ/kg and 30.1 MJ/kg). The heating value is positively correlated with the fixed carbon. The extension of the carbonization process determines an increase in fixed carbon and consequently also in the heating value [23]. The European samples have higher values of fixed carbon than the other groups. Moreover, the European samples have shown an ash content lower than the other ones. The presence, in fact, of a higher content of elements not participating in the combustion process determines a reduction of the heating value [43]. This is especially evident for the group of charcoal briquettes. The average value found with the standard method is 24.8 MJ/kg and 24.1 MJ/kg with the proposed method. With both methods the values are much lower than what is observed in the charcoal [44]. This is linked to the higher ash content of the briquettes. Low energy values in the charcoal briquettes were also found by Dias Júnior et al. [22], ranging between 17.4 and 23.0 MJ/kg.

**Figure 6:**
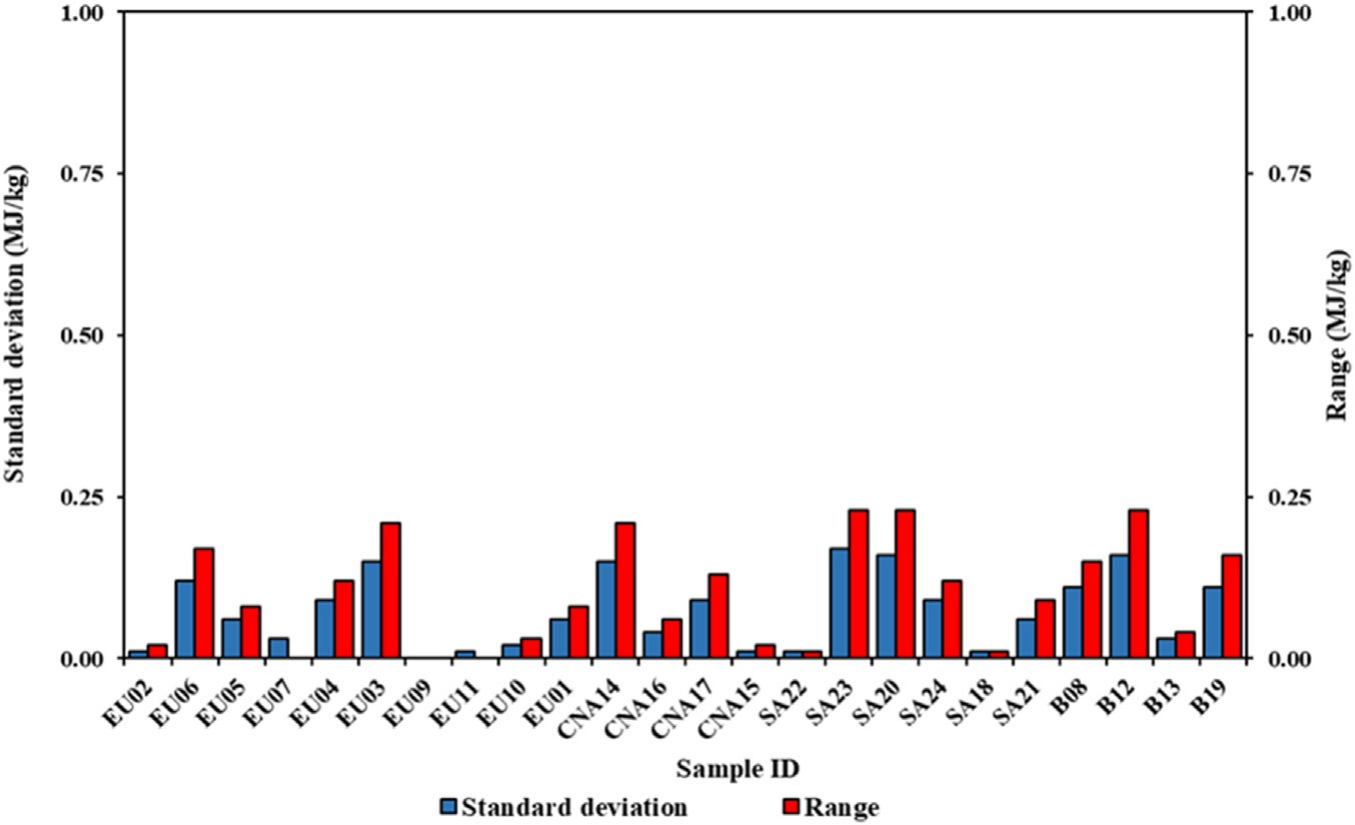
Variability of the HHV_0_ of all samples in terms of standard deviation and range determined according to the standard method.

**Figure 7:**
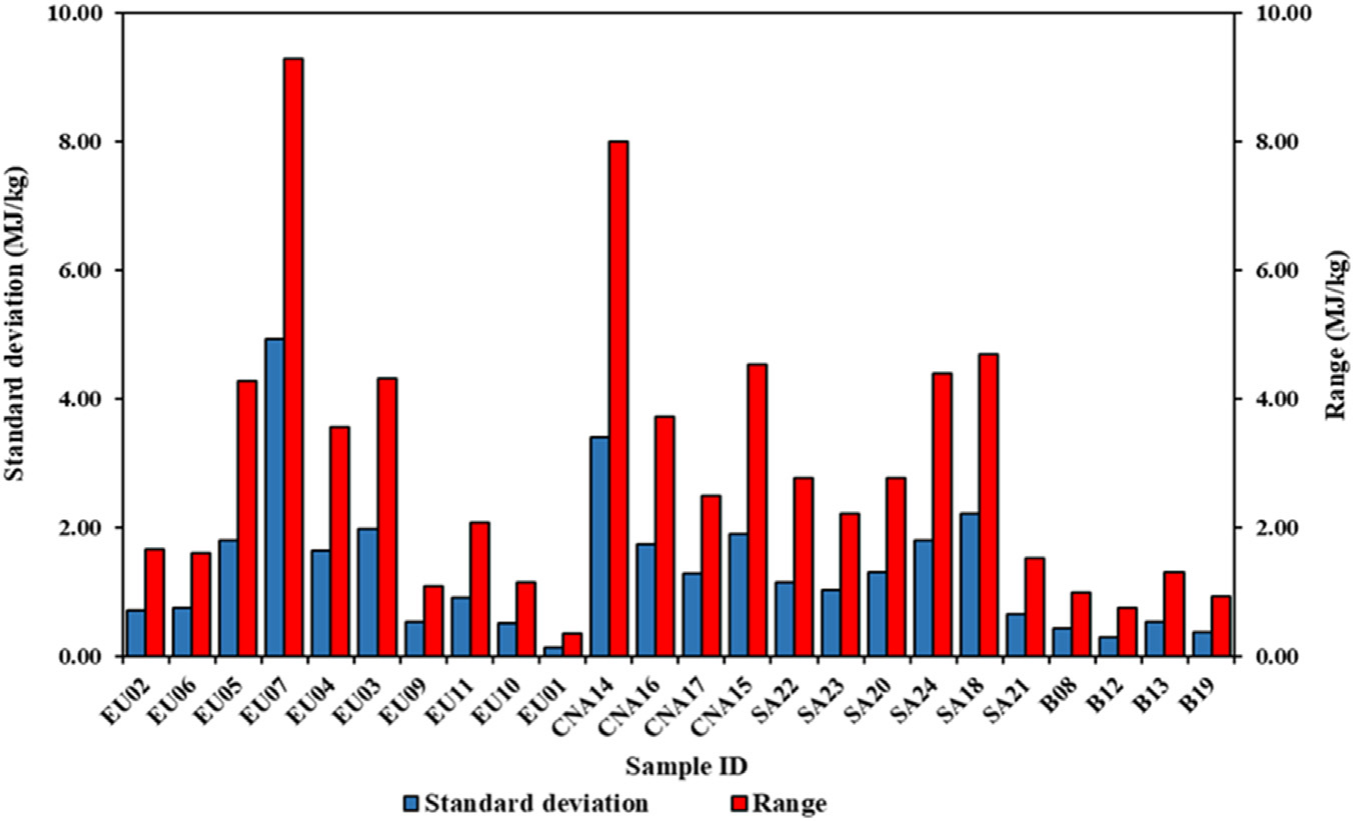
Variability of the HHV_0_ of all samples in terms of standard deviation and range determined according to the proposed method.

Comparing the results determined with both methods, regarding the repeatability of the individual tests. The standard method shows great homogeneity and repeatability (Figure 6). The standard deviation values for all samples are contained below the threshold of 0.20 MJ/kg. Seventeen of the twenty-four analyzed samples have a value lower than 0.10 MJ/kg. The range of variations also has limited oscillations. The maximum value detected was of 0.17 MJ/kg referring to the SA23 sample. Finally, sixteen of the twenty-four samples have repeatability values lower than those required by EN ISO 18125:2017 of 0.14 MJ/kg [28]. Instead, with reference to the proposed method, a low homogeneity found in the single calorimetric tests is highlighted. In this case the standard deviation and range values are significantly higher (Figure 7). For the standard deviation the lowest determined value is 0.15 MJ/kg, in the EU10 sample. Most of the samples show significantly higher values, even exceeding 2.00 MJ/kg as found in the samples EU07, CNA14 and SA18. The range of variation has also increased significantly. Fluctuations over 9.00 MJ/kg were found. The low energy homogeneity of the single subsamples does not allow to obtain the same repeatability of the standard method. Consequently, none of the samples analyzed with the proposed method falls within the repeatability limit of the standard.

## 4 Conclusions

This work highlights the scarce homogeneity in terms of the energy properties of barbecue charcoal commonly found on the Italian market. The analysis conducted, according to the reference standard, show how the characteristics of the material are significantly influenced by origin, trees and production process used. The European samples show better qualitative parameter values than the other groups. This is linked to the characteristics of the raw wood material and the carbonization process involved [36]. In these samples the highest values of heating value (32.3 MJ/kg-33.2 MJ/kg) and fixed carbon (77.2%) were determined, showing at the same time low values of ashes (2.9%) and volatile matter (14.8%). As regard the American groups, both have a higher value of ash (CNA 8.4%) (SA 7.3%) and volatile matter (CNA 24.5%) (SA 22.9%) and at the same time lower values of fixed carbon (CNA 62.0%) (SA 64.1%) than European samples. As already highlighted by Dias Júnior et al. [22], briquettes have lower quality characteristics than charcoal for barbecues uses. These have high values of ash content (17.6%), moisture content (7.0%), volatile matter (24.7%) and low values of fixed carbon (50.0%). Improvements in current standards are also necessary. The HHV_0_ determined applying the standard method is highly homogeneous and repeatable but may not reflect the energetic properties of the material. Therefore, to improve the representativeness of the value found, it is necessary to link the average HHV_0_ value determined following the standard procedure with the range of values found on unground material [29]. Finally, the standard does not require any chemical analyses of the material. However, these could be useful to deepen the knowledge on the characteristics of the material with particular attention to the presence of metals, harmful to human health [45]. Therefore, it is of primary importance to highlight the qualitative characteristics of the material used for cooking food and to get to the definition of a quality brand. This to ensure the consumers to buy products with controlled quality and at the same time respectful of the environment.

## Declarations

### Author contribution statement

Mencarelli, A: Performed the experiments; Analyzed and interpreted the data; Contributed reagents, materials, analysis tools or data; Wrote the paper.

Cavalli, R: Conceived and designed the experiments; Wrote the paper.

Greco, R: Conceived and designed the experiments; Analyzed and interpreted the data; Contributed reagents, materials, analysis tools or data; Wrote the paper.

### Funding statement

This research did not receive any specific grant from funding agencies in the public, commercial, or not-for-profit sectors.

### Data availability statement

Data will be made available on request.

### Declaration of interests statement

The authors declare no conflict of interest.

### Additional information

No additional information is available for this paper.
